# Construction of the prognostic signature of alternative splicing revealed the prognostic predictor and immune microenvironment in head and neck squamous cell carcinoma

**DOI:** 10.3389/fgene.2022.989081

**Published:** 2022-10-21

**Authors:** Fan Ye, Pingan Wu, Yaqiong Zhu, Guan Huang, Ying Tao, Zhencheng Liao, Yafeng Guan

**Affiliations:** ^1^ Department of Surgery, Division of Otolaryngology, Head and Neck Surgery, The University of Hong Kong-Shenzhen Hospital, Shenzhen, China; ^2^ Department of Otolaryngology Head and Neck Surgery, Second Affiliated Hospital of Nanchang University, Nanchang, China

**Keywords:** head and neck squamous cell carcinoma, alternative splicing, prognostic signature, immune microenvironment, risk score

## Abstract

**Background:** Head and neck squamous cell carcinoma (HNSC) is a prevalent and heterogeneous malignancy with poor prognosis and high mortality rates. There is significant evidence of alternative splicing (AS) contributing to tumor development, suggesting its potential in predicting prognosis and therapeutic efficacy. This study aims to establish an AS-based prognostic signature in HNSC patients.

**Methods:** The expression profiles and clinical information of 486 HNSC patients were downloaded from the TCGA database, and the AS data were downloaded from the TCGA SpliceSeq database. The survival-associated AS events were identified by conducting a Cox regression analysis and utilized to develop a prognostic signature by fitting into a LASSO-regularized Cox regression model. Survival analysis, univariate and multivariate Cox regression analysis, and receiver operating characteristic (ROC) curve analysis were performed to evaluate the signature and an independent cohort was used for validation. The immune cell function and infiltration were analyzed by CIBERSORT and the ssGSEA algorithm.

**Results:** Univariate Cox regression analysis identified 2726 survival-associated AS events from 1714 genes. The correlation network reported DDX39B, PRPF39, and ARGLU1 as key splicing factors (SF) regulating these AS events. Eight survival-associated AS events were selected and validated by LASSO regression to develop a prognostic signature. It was confirmed that this signature could predict HNSC outcomes independent of other variables *via* multivariate Cox regression analysis. The risk score AUC was more than 0.75 for 3 years, highlighting the signature’s prediction capability. Immune infiltration analysis reported different immune cell distributions between the two risk groups. The immune cell content was higher in the high-risk group than in the low-risk group. The correlation analysis revealed a significant correlation between risk score, immune cell subsets, and immune checkpoint expression.

**Conclusion:** The prognostic signature developed from survival-associated AS events could predict the prognosis of HNSC patients and their clinical response to immunotherapy. However, this signature requires further research and validation in larger cohort studies.

## Introduction

Head and neck squamous cell carcinoma (HNSC) is the sixth most common cancer globally, with an estimated 900,000 new cases and 450,000 deaths annually (Ferlay et al., 2019). HNSC patients are normally diagnosed at the later stages with a poor prognosis. Although chemotherapy, radiation, and targeted therapies have made great progress, the survival rate of advanced or recurrent HNSC remains low (Cohen et al., 2016). This is due to the heterogeneity of HNSC. Patients with advanced HNSC are treated with cetuximab, an anti-EGFR antibody, with a 13% success rate (Licitra et al., 2011). An immunotherapy agent, anti-PD1 antibody, successfully stimulated anti-tumor immunity and produced a significant clinical response in patients with aggressive HNSC. However, only a subset of patients (∼18%) benefitted from this strategy, while most HNSC patients displayed clinical resistance (Chow et al., 2016; Ferris et al., 2016; Seiwert et al., 2016). Therefore, it is crucial to identify new markers to provide an accurate HNSC prognosis and the appropriate treatment strategies.

Alternative splicing (AS) is a process in which mRNA precursors are alternatively spliced and ligated to produce mature mRNAs for protein diversity (Larochelle, 2016; Ule and Blencowe, 2019). Research has identified the contribution of AS to a variety of diseases, including cancer. The dysregulated expression of splice isoforms is considered a potential driver of tumor development and progression (Sciarrillo et al., 2020). Recent studies suggest that cancer-specific splice isoforms can be important signatures for predicting treatment efficacy. For instance, squamous cell carcinoma patients with EGFR isoform D splicing patterns showed better responses toward EGFR-TKIs (Tan et al., 2017). More importantly, AS was reported to contribute to the development of the immune microenvironment (Frankiw et al., 2019; Li et al., 2019; Yu et al., 2020). Changes in AS can alter both immune cell infiltration and tumor-associated immune cytolytic activity. Hence, AS signature may accurately predict the clinical response of HNSC patients to immunotherapy.

Herein, a comprehensive analysis of genome-wide AS events was performed with RNA sequencing (RNA-Seq) data obtained from TCGA-HNSC samples. We developed a novel prognostic signature from the data *via* LASSO regression analysis. HNSC patients could be categorized into either high or low-risk groups based on their risk scores calculated with the prognostic signature. Immune infiltration and functional analysis were then performed to identify the role of the signature in the tumor microenvironment. Our analysis suggested that this prognostic signature could be an effective tool for predicting the prognosis of HNSC patients and their clinical response to immunotherapy.

## Methods

### Data collection and pretreatment

**FIGURE 1:**
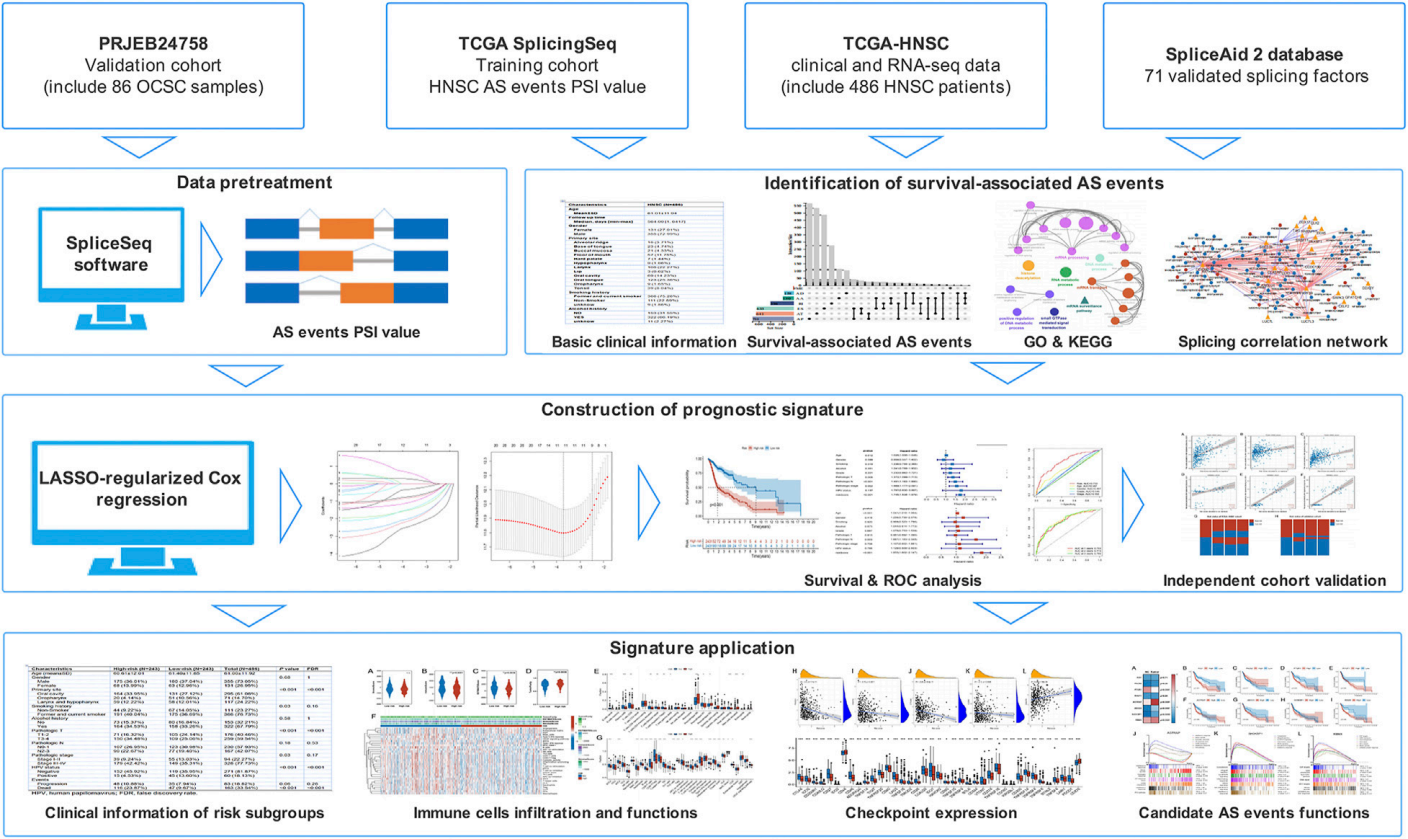
The flow chart of AS-based prognostic signature construction and evaluation.

The expression profiles, somatic mutation, and matching clinical follow-up information of HNSC patients were obtained from the TCGA database. AS data was obtained from the TCGA SpliceSeq database. A total of 486 patients were enrolled based on the following criteria (Ferlay et al., 2019): histologically confirmed HNSC (Cohen et al., 2016); patients with RNA-Seq data; and (Licitra et al., 2011) patients with basic clinical and follow-up information. Patients who lacked AS data in the TCGA SpliceSeq database had been excluded. HPV status information was collected from published data and the TCGA database (Cancer Genome Atlas Network, 2015). The clinical characteristics of patients are listed in Supplementary Table S1. For validation, RNA-Seq data of an independent cohort (86 oral cavity squamous cell carcinoma patients) was accessed from the European Nucleotide Archive (study accession: PRJEB24758) and processed by SpliceSeq software to obtain the AS profiles. The percent spliced-in index (PSI), a ratio of normalized read counts indicating the inclusion of a transcript element over the total normalized reads, was used to quantify AS events (Ryan et al., 2012). From here, seven different types of AS events were identified: exon skip (ES), alternate donor site (AD), alternate acceptor site (AA), retained intron (RI), exclusive exons (ME), alternate terminator (AT), and alternate promoter (AP). To obtain a set of more reliable AS events, several conditions were implemented (i.e., percentage of samples with PSI value ≥75, average PSI value ≥0.05). Figure 1 depicts the flow chart representative of this study.

### Identification and functional analysis of survival-associated AS events

The hazard ratios (HRs) and 95% confidence interval (95% CI) of the overall survival of AS events were calculated after performing a univariate Cox regression analysis. AS events with *p* < 0.05 were considered survival-associated AS events. The seven types of identified AS events were utilized to develop an UpSet plot with a “UpSetR” package (version 1.4.0). Gene Ontology (GO) and Kyoto Encyclopedia of Genes and Genomes (KEGG) analysis were performed to determine the parental genes in survival associated AS events. To visualize the results, functionally organized networks were constructed through the Cytoscape plug-in, ClueGO (version 2.5.8), with network connectivity (κ-score) ≥ 0.5, as described by Bindea et al. (Bindea et al., 2009).

### Splicing correlation network construction

The list of 71 experimentally validated splicing factor (SF) genes was obtained from the SpliceAid 2 database, and their expression profiles were obtained from the TCGA database. Pearson correlation analysis was performed between SF expression and PSI value of survival-associated AS events, with several conditions (i.e., |R| ≥ 0.7, and *p* < 0.001). The splicing correlation network was then established using the Cytoscape software (version 3.8.2).

### Construction and evaluation of the prognostic signature

Our prognostic signature was developed based on a LASSO-regularized Cox regression model (Luo et al., 2020; Wang et al., 2021a; Guo et al., 2021). The survival-associated AS events obtained above were input into the LASSO regression analysis with a “glmnet” package (version 4.1.3) to remove highly correlated variables and prevent overfitting. The optimum shrinkage parameter (*λ*) was determined using 10-fold cross-validation. AS events with non-zero parameters estimated in the model were selected and used to fit a multivariate Cox regression model to obtain the regression coefficients. The risk score of each sample was calculated by the formula: $Risk\;Score=\overset{n}{\underset{i=1}{\sum }}{Coef}_{i}\times {PSI}_{i}$ , where ${Coef}_{i}$ is the coefficient and ${PSI}_{i}$ is the PSI value of selected AS events.

From the median risk score, patients were classified into two risk groups. Kaplan–Meier survival analysis with a log-rank test was conducted to verify the statistical differences. Then, each selected AS event’s risk score distribution, survival time, and expression thermogram were visualized. Univariate and multivariate Cox regression models were fitted to analyze whether the risk scores were independent of clinical variables. A time-dependent receiver operating characteristic (ROC) curve analysis was performed to evaluate the prediction capability of the prognostic signature.

Validation of the prognostic signature was performed with an indirect method due to insufficient RNA-Seq data with survival information. Briefly, three published and well-validated prognostic signatures served as references and were used to calculate risk scores for the TCGA-HNSC and independent cohort samples (Wang et al., 2021c; Liu et al., 2021a; Chen et al., 2022). Our AS-based signature was also used to score the patient with the PSI profiles produced by SpliceSeq software. Correlation analysis and heatmaps were then used to demonstrate the consistency between the calculated risk scores of the prognostic signature and other signatures.

### Immune cell infiltration and functional analysis

We used ESTIMATE, CIBERSORT, and ssGSEA to measure immune cell infiltration and functional differences between the risk subgroups. The presence of infiltrating stromal and immune cells in tumor samples was estimated with the “estimate” package (version 1.0.13) and classified into four scores: Stromal, Immune, ESTIMATE, and Tumor purity. The ESTIMATE score that infers tumor purity is the sum of immune and stromal enrichment scores and can be converted to tumor purity score as previously mentioned (Yoshihara et al., 2013). Immune cell infiltration in each sample was quantified by CIBERSORT, a validated deconvolution algorithm for characterizing cell composition based on the leukocyte signature matrix (LM22) (Newman et al., 2015). We used an R script to implement this algorithm with 1000 permutations, without quantile normalization. The quantified immune population was validated with ssGSEA using the “GSVA” package (version 1.40.1). The immune signatures that were identified using this approach included immune cell types (e.g., plasmacytoid DCs, inactivated DCs, activated DC, DCs, NK cells, B cells, mast cells, neutrophils, macrophages, CD8^+^ T cells, helper T-cells, Tfh, Th1, Th2, and Treg), immune-related functions (e.g., APC co-inhibition, APC co-stimulation, T cell co-inhibition, T cell co-stimulation, cytolytic activity, type I IFN response, type II IFN response, pro-inflammation, HLA, MHC class I, and CCR), and immune-related pathways (e.g. extracellular matrix, epithelial-mesenchymal transition, angiogenesis, and VEGF signaling pathway). These signatures were collected from publications and the MsigDB database (Barbie et al., 2009; Xue et al., 2019). A heatmap and a boxplot were plotted to visualize these results with the “heatmap” (version 1.0.12) and “ggplot2” (version 3.3.5) packages. The differential expression analysis for the identification of immune checkpoints between the two groups was performed with the “limma” package (version 3.48.3) and the Wilcoxon test. The correlation coefficient between risk score and immune checkpoint expression was calculated with Spearman correlation analysis. The tumor mutational burden (TMB) in each HNSC patient was calculated with the “maftools” package (version 1.0.12) from the somatic mutation data downloaded from the TCGA.

### Functional analysis of the AS parent genes involved in the prognostic signature

We performed expression, survival, and functional analysis to determine the AS parent genes. For expression analysis, the log2-transformed Fragments Per Kilobase Million (FPKM) value of each gene was counted, and the differences between tumor and normal groups were analyzed by the GraphPad 8.0 software and the Mann-Whitney test. For survival analysis, HNSC patients were categorized into two groups based on their gene expression. The survival outcomes were determined through a Kaplan-Meier analysis. The pathways that were significantly linked to the expression of parent genes were identified with GSEA analysis (version 4.1.0) of the Hallmark gene sets. Using the gene expression lists ranked by Pearson, we calculated the weighted enrichment scores. Gene sets with a nominal *p*-value < 0.05 and FDR ≤0.1 were considered statistically significant. The first seven significantly correlated pathways were visualized using GraphPad 8.0 software. For immune function analysis, Spearman’s correlation method was used to calculate the correlation coefficient between gene expression and immune checkpoint or immune cell infiltration. The immune correlation network was then established using the Cytoscape software (version 3.8.2) with a *p*-value < 0.05 threshold.

### Statistical analyses

R (version 4.1.0) and GraphPad 8.0 were utilized for all statistical analyses. *p*-value < 0.05 was statistically significant. The categorical clinical characteristics variables were analyzed by the Chi-square and Fisher’s exact tests, while the continuous variables were analyzed by the Mann-Whitney non-parametric and ANOVA tests. The non-normal data distribution was analyzed *via* Spearman correlation analysis, while the continuous variables with normal distribution were analyzed *via* the Pearson correlation analysis.

## Result

### Identification of survival-associated AS events in HNSC

**FIGURE 2:**
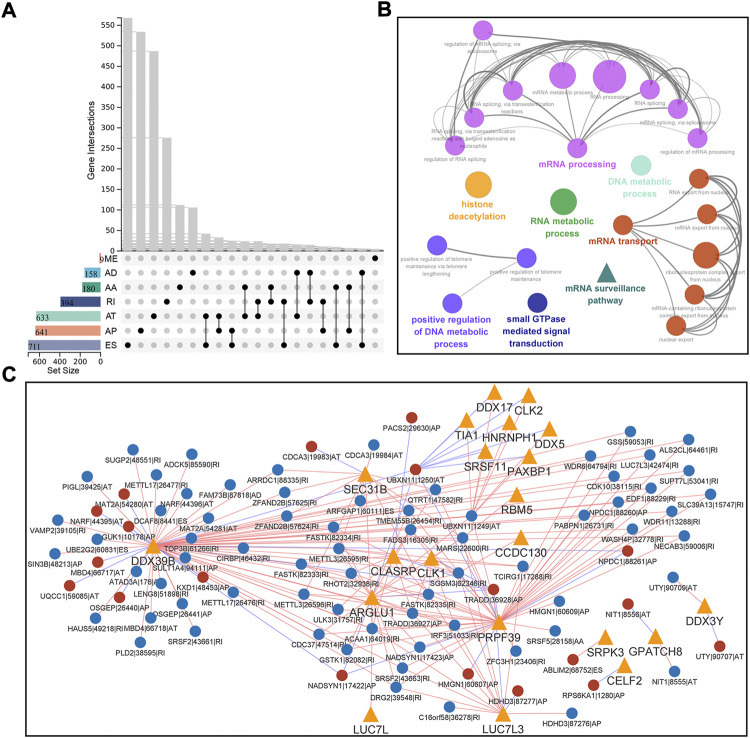
Identification and functional analysis of survival-associated AS events in HNSC. **(A)** Interactive sets between the seven types of survival-associated AS events (*n* = 2726) shown in an UpSet plot. **(B)** Functionally grouped Gene Ontology (GO) and Kyoto Encyclopedia of Genes and Genomes (KEGG) categories for parent genes of survival-associated AS events. GO and KEGG categories were grouped based on their similarity (κ-score ≥ 0.5), and the most significant term in each group is shown in bold. **(C)** Correlation network of splicing factors and survival-associated AS events. This network was built based on significant correlations (|R| ≥ 0.7, *p* < 0.001) between the expression of 71 splicing factors and the PSI values of survival-associated AS events. Splicing factors are represented with orange triangles, and AS events are represented with circulars (red/blue represents favorable/inferior prognosis). The red/blue lines are represented with positive/negative correlation. Data were analyzed using Pearson’s correlation method.

Univariate Cox regression analysis were done on 486 HNSC patients to evaluate the correlation between AS events and OS. Out of 1714 genes, 2726 AS events were identified as survival-associated. ES was the predominant type (26.1%), followed by AP (23.5%) and AT (23.2%). Given the multiple splicing patterns for a single gene, the genes intersecting sets and survival-associated AS events were visualized using the UpSet plot (Figure 2A). Function enrichment analyses were performed to study the biological characteristics of AS events. Our findings revealed that the parent genes were enriched in KEGG and BP, which were related to important biological functions, including mRNA surveillance pathway, adherens junction, RNA transport, protein localization, intracellular transport, and mRNA metabolic process (Figure 2B).

Splicing factors (SFs) regulate AS events. To better understand the regulation of survival-associated AS events, we performed a Pearson correlation analysis between reported 71 expressions of SFs and PSI values. Following the significant correlation (|r|≥0.7, *p* < 0.001), a splicing regulatory network was built, which contained 206 SF-AS pairs, 21 SFs, and 88 survival-associated AS events (Figure 2C). Most SF-AS pairs were positively correlated in this network. Splicing factors such as DDX39B, PRPF39, and ARGLU1 might be key regulators in AS events.

### The construction of the prognostic signature of AS events revealed the prognostic predictor in HNSC

**FIGURE 3:**
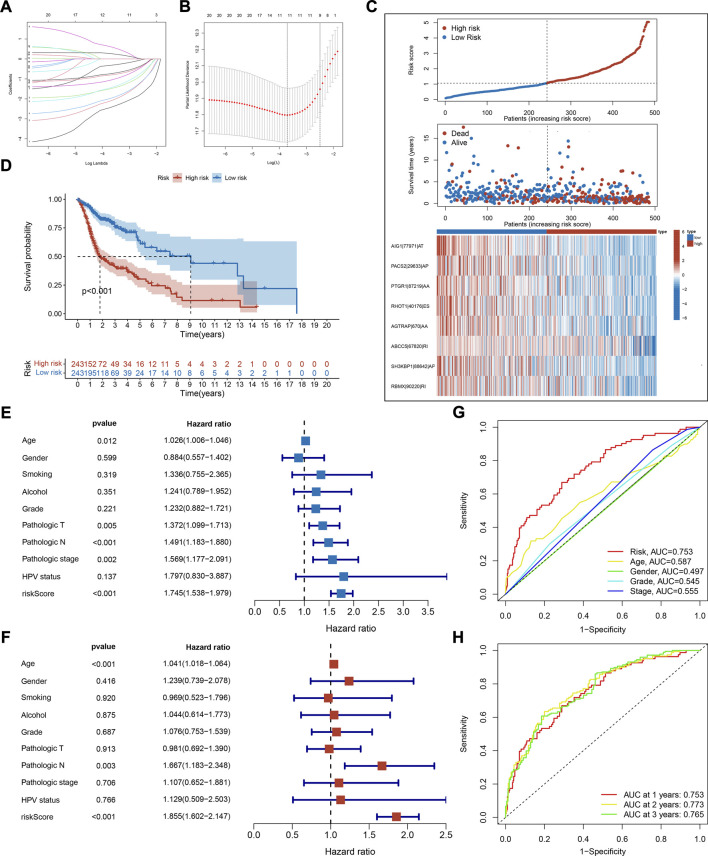
Construction and evaluation of prognostic signature. **(A)** Distribution of LASSO regression coefficients for survival-associated AS events. **(B)** Shrinkage parameter selection in the LASSO model used ten fold cross-validation *via* minimum criteria. Vertical dotted lines were drawn at the optimum *λ* values. **(C)** The risk score distribution, overall survival status, and expression profile of selected AS events of each HNSC sample. **(D)** Kaplan-Meier survival curve of overall survival. Log-rank test was used for data analysis. **(E,F)** Forest plots of hazard ratios (HRs) calculated by univariate **(E)** and multivariate **(F)** Cox regression for risk score and clinical features associated with overall survival. **(G)** Receiver operating characteristic (ROC) curve and the area under the curve (AUC) of risk score and clinical features in predictive performance for HNSC patients. **(H)** ROC plots and AUC of the risk score at 1, 2, and 3 years.

Survival-associated AS events were fitted to LASSO Cox regression analysis to remove highly correlated variables and prevent overfitting when constructing the prognostic signature. The shrinkage parameter was determined using 10-fold cross-validation. This signature achieved minimal deviation with 11 OS-associated AS events, but only eight of them were retained after optimization by a stepwise multivariate Cox regression analysis (Figures 3A,B). The details of these AS events, including their corresponding coefficients and hazard ratios are listed in Supplementary Table S2. The risk scores of HNSC patients were calculated according to the PSI values and their coefficients as follows: riskscore=−3.77×AIG1|77971|AT−2.71×PACS2|29633|AP−2.20×PTGR1|87219|AA−2.46×RHOT1|40176|ES−1.98×AGTRAP|670|AA−0.98×ABCC5|67820|RI−1.45×SH3KBP1|88642|AP−1.02×RBMX|90220|RI .

To study the correlation between risk scores and clinical features, HNSC patients were categorized into two risk groups. The clinical characteristics of these groups are shown in Table 1. Notably, there were more HPV-positive patients in the low-risk group. The score distribution, overall survival status, and expression profile of AS events are as plotted in Figure 3C. The high-risk group had a lower OS rate as the Kaplan-Meier survival analysis indicated (*p* < 0.001, Figure 3D), which validated the prediction capability of our prognostic signature. To establish that risk score is independent of other variables in predicting HNSC outcome, univariate and multivariate Cox regression analyses were employed using clinical features like age, gender, pathologic stage, and HPV status. The analysis reported that the risk score variable was statistically significant (Figures 3E,F). From the ROC analysis, the AUC of risk score was higher than the other variables and remained above 0.75 for 3 years, which suggested a powerful predicting capability of our signature (Figures 3G,H). Together, these results revealed the satisfactory efficiency of our signature in predicting prognosis for HNSC patients.

**TABLE 1:** Correlation between risk score based subgroups and clinical characteristics.

Characteristics	High-risk (*n* = 243)	Low-risk (*n* = 243)	Total (*n* = 486)	*p* Value	FDR
Age (mean ± SD)	60.61 ± 12.01	61.40 ± 11.85	61.00 ± 11.92		
Gender				0.68	1
Male	175 (36.01%)	180 (37.04%)	355 (73.05%)		
Female	68 (13.99%)	63 (12.96%)	131 (26.95%)		
Primary site				<0.001	<0.001
Oral cavity	164 (33.95%)	131 (27.12%)	295 (61.08%)		
Oropharynx	20 (4.14%)	51 (10.56%)	71 (14.70%)		
Larynx and hypopharynx	59 (12.22%)	58 (12.01%)	117 (24.22%)		
Smoking history				0.03	0.16
Non-Smoker	44 (9.22%)	67 (14.05%)	111 (23.27%)		
Former and current smoker	191 (40.04%)	175 (36.69%)	366 (76.73%)		
Alcohol history				0.58	1
No	73 (15.37%)	80 (16.84%)	153 (32.21%)		
Yes	164 (34.53%)	158 (33.26%)	322 (67.79%)		
Pathologic T				<0.001	<0.001
T1-2	71 (16.32%)	105 (24.14%)	176 (40.46%)		
T3-4	150 (34.48%)	109 (25.06%)	259 (59.54%)		
Pathologic N				0.18	0.53
N0-1	107 (26.95%)	123 (30.98%)	230 (57.93%)		
N2-3	90 (22.67%)	77 (19.40%)	167 (42.07%)		
Pathologic stage				0.03	0.17
Stage I-II	39 (9.24%)	55 (13.03%)	94 (22.27%)		
Stage III-IV	179 (42.42%)	149 (35.31%)	328 (77.73%)		
HPV status				<0.001	<0.001
Negative	152 (45.92%)	119 (35.95%)	271 (81.87%)		
Positive	15 (4.53%)	45 (13.60%)	60 (18.13%)		
Events					
Progression	48 (10.88%)	35 (7.94%)	83 (18.82%)	0.06	0.26
Dead	116 (23.87%)	47 (9.67%)	163 (33.54%)	<0.001	<0.001

HPV, human papillomavirus; FDR, false discovery rate.

**FIGURE 4:**
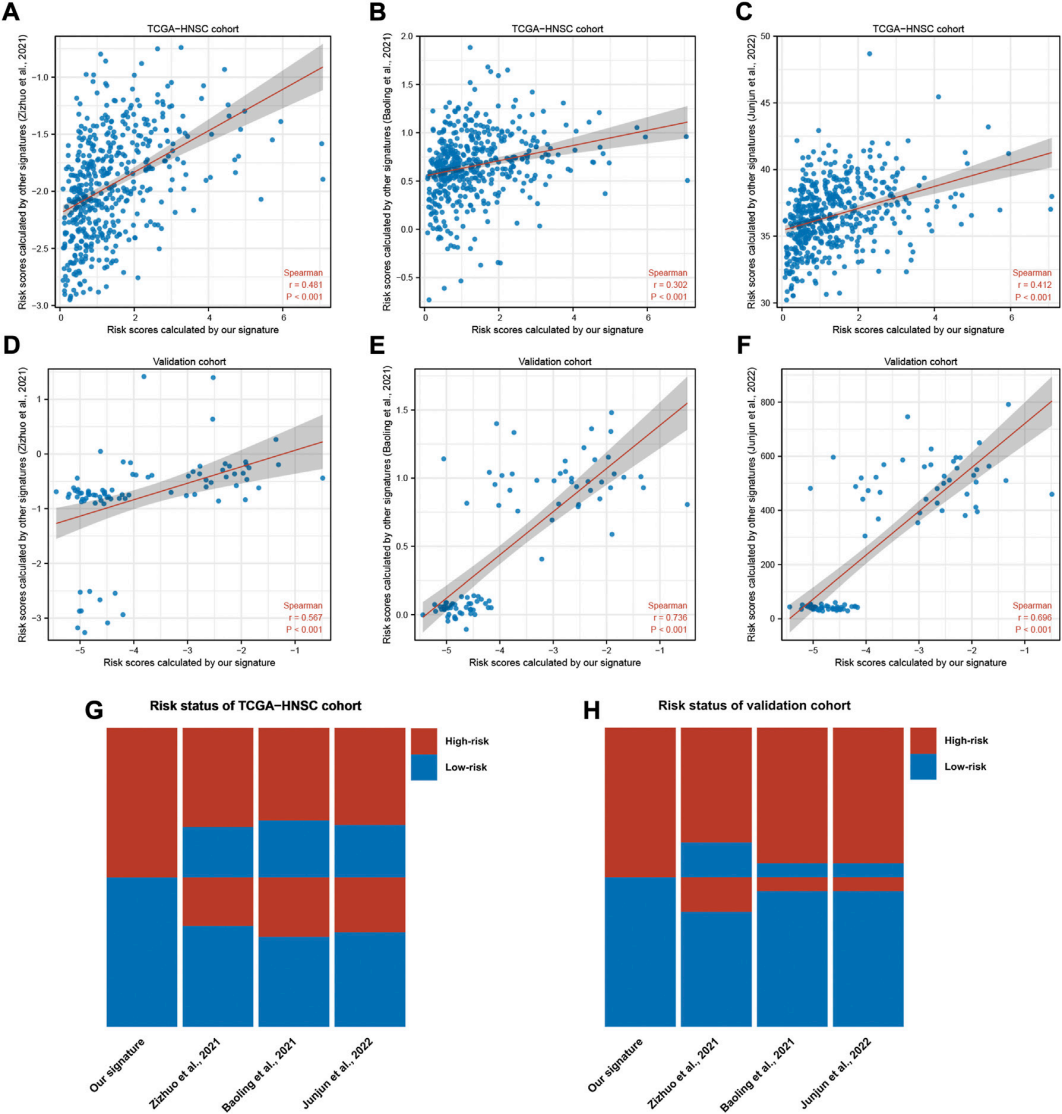
Validation of our signature in a independent HNSC cohort. **(A–F)** Scatter plots of correlations between the risk scores calculated by our signature and that calculated by reference signatures in TCGA-HNSC and independent cohort. Data were analyzed using Spearman’s correlation method. **(G,H)** Heatmaps showing the risk status of each sample predicted by our signature and reference signatures.

To validate our signature in an independent cohort, we utilized three published and well-validated prognostic signatures as references in our study. Correlation analysis reported that risk scores correlated significantly with the reference signatures in the TCGA-HNSC and independent cohorts (Figures 4A–F). The risk status of HNSC patients was visualized with a heatmap, revealing the consistency of our signature with the reference signatures in predicting the prognosis of HNSC (Figures 4G,H). These results validated the credibility of our prognostic signature.

### Correlation of risk score with the immune microenvironment signatures

**FIGURE 5:**
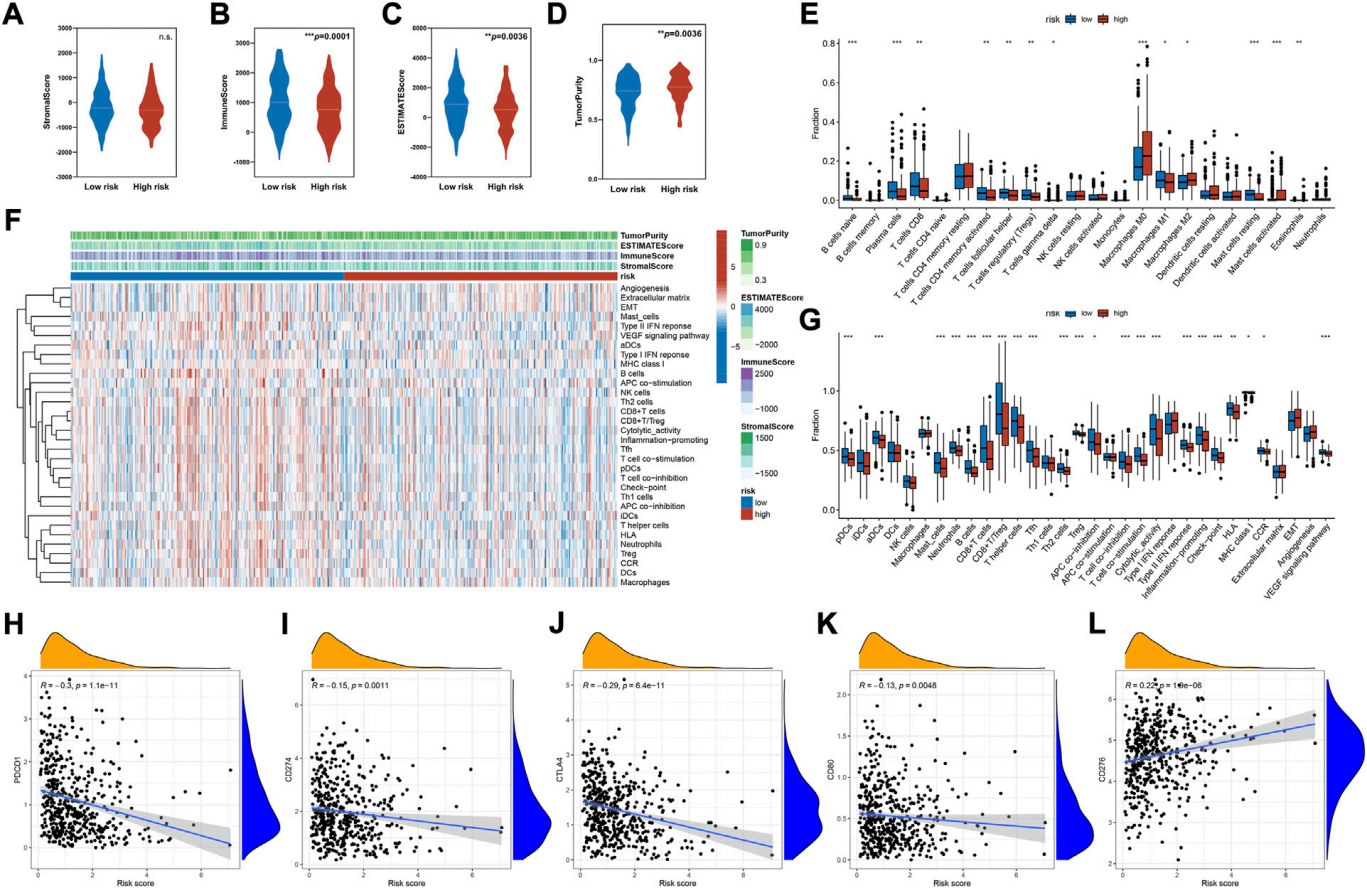
Immune cells infiltration and functional analysis. **(A–D)** The analysis of the stromal score, immune score, ESTIMATE score, and tumor purity between the high- and low-risk groups. **(E)** Immune cell subpopulations analysis between the high- and low-risk groups based on CIBERSORT algorithm. **(F)** Heatmap of the enrichment score of immune cells subpopulations and immune-related pathways signatures based on ssGSEA algorithm. **(G)** Differential enrichment analysis of immune cells subpopulations and immune-related pathways between the high- and low-risk groups based on ssGSEA algorithm. **(H–L)** Scatter plot of correlations between representative immune checkpoint expression and risk score. Data were analyzed using Spearman’s correlation method. *p < 0.05, **p < 0.01, ***p < 0.001, ****p < 0.0001.

Given the pivotal role of the immune microenvironment in tumor development and progression, we investigated the correlation between risk score and immune features. As shown in Figures 5A–D, the immune and stromal cells in tumors were quantified based on the ESTIMATE algorithm. This finding indicated that the high-risk group had lower immune and stromal cell concentration and higher tumor purity (Figures 5A–D). Immune subpopulation analysis demonstrated that tumors in the high-risk group were highly infiltrated with immune cells such as M2 and M0 macrophages, and lowly infiltrated with CD8^+^ T cells, Treg, memory CD4 T cells, naïve B cells, and M1 macrophages (Figure 5E). Further correlation analysis indicated that the infiltration of activated mast cells, eosinophils, M2 macrophages, and M0 macrophages had a positive correlation with the risk score. On the other hand, naïve B cells, plasma cells, T follicular helper cells, Tregs, resting mast cells, CB8+ T cells, memory CD4 T cells, and γδ T cells had a negative correlation with the risk score (Supplementary Figure S1A, ranked by the Spearman correlation coefficients). Related immune function signatures were quantified using ssGSEA analysis and visualized with a heatmap (Figures 5F,G). The result was consistent with the distribution of immune cell subpopulations, where the immune functions such as cytolytic activity, T cell co-stimulation, and APC co-stimulation were lower in the high-risk group. We also analyzed the expression of immune checkpoints and demonstrated that most immune checkpoints were lowly expressed in the high-risk group (Supplementary Figure S1B). The correlation analysis indicated that the risk scores were negatively correlated with PDCD1, CD274, CTLA4, and CD80, and positively correlated with CD276 (Figures 5H–L). TMB was linked to immune infiltration of tumors (Goodman et al., 2017; Jardim et al., 2021), but no significant difference was found between the two groups (Supplementary Figure S1C).

### AS events involved in our signature might play a role in the HNSC tumorigenesis, prognosis, and immune regulation.

**FIGURE 6:**
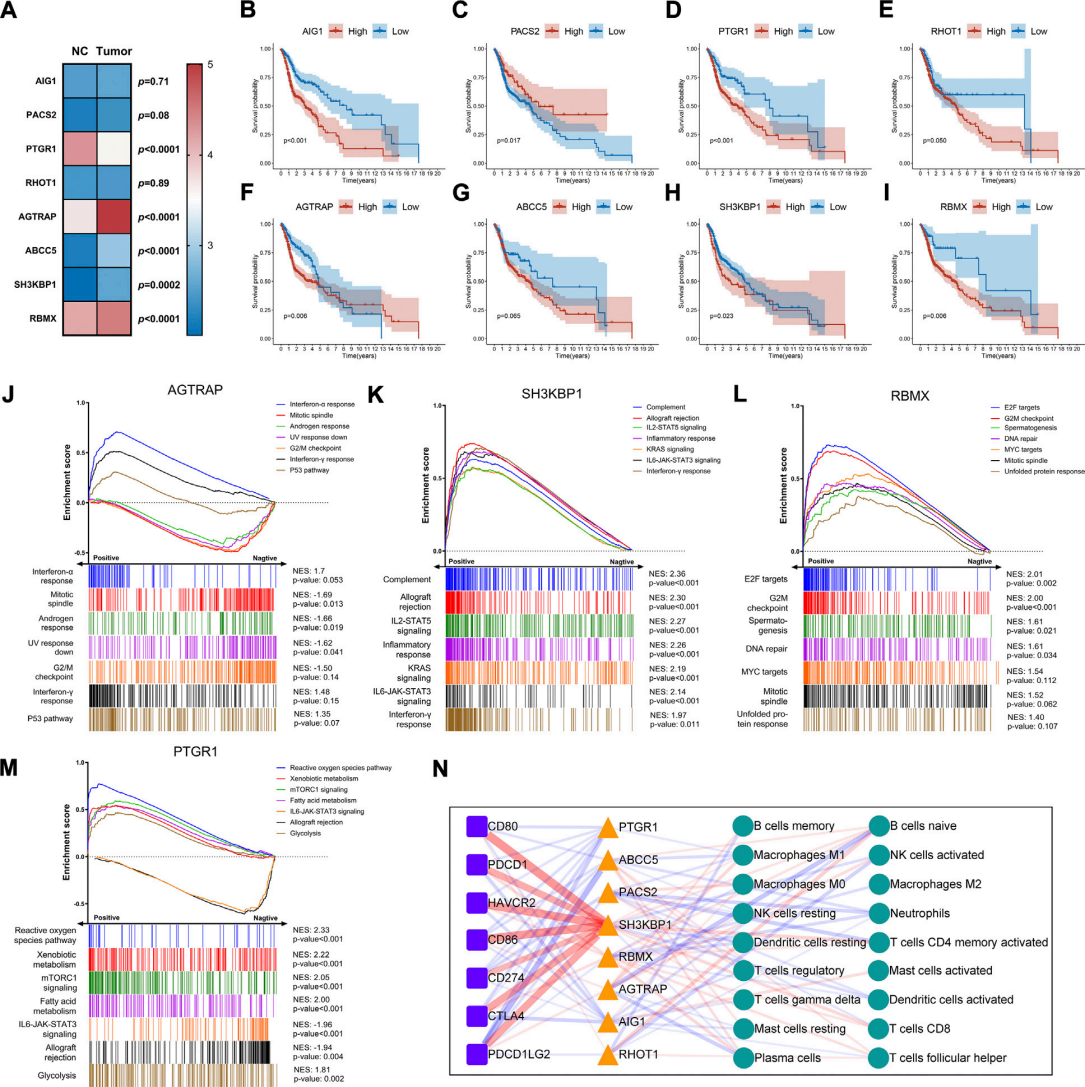
Expression and functional analysis of the parent genes of AS involved in prognostic signature. **(A)** Heatmap and differential expression analysis of the parent genes between HNSC and normal samples. The filled color represents the log2 (FPKM value) for each gene. Data were analyzed using Mann-Whitney nonparametric test. **(B–I)** Kaplan-Meier survival curve of overall survival for each parent gene. Log-rank test was used for data analysis. **(J–M)** GSEA plots for the top seven Hallmark gene sets significantly correlated with the parent gene expression. The enrichment scores were calculated with gene expression lists ranked by pearson. NES, normalized enrichment score. **(N)** The network diagram shows the correlation between parent genes, immune checkpoints, and immune cell subsets (CIBERSORT). Colored triangles, squares, and circles represent respectively parent genes, immune checkpoints, and immune cells, respectively. The thickness of the line represents the strength of correlation. Red and blue represent positive and negative correlation, respectively. Data were analyzed using Spearman’s correlation method.

We studied the expression, function, and prognosis of parent genes in AS events. Expression analysis indicated that five genes had significant differential expressions between tumor and normal samples (Figure 6A). AGTRAP1, ABCC5, SH3KBP1, and RBMX were upregulated, while PTGR1 was downregulated in tumor samples as compared to normal samples (Figure 6A). Survival analysis showed that AIG1, PACS2, PTGR1, AGTRAP, SH3KBP1, and RBMX expressions significantly affected the overall survival (Figures 6B–I). High expressions of AGTRAP, SH3KBP1, and RBMX were found in tumor tissues and correlated with a poor prognosis. This finding suggested that these genes could have a role in tumor initiation and progression. The lack of correlation between the PTGR1 expression and prognosis suggested its complex regulatory mechanism. We then analyzed the pathways and functions of these genes using GSEA with Hallmark gene sets. The seven significantly enriched gene sets are shown in Figures 6J–M. These results revealed that these parent genes could be involved in HNSC development. Notably, SH3KBP1 had a significantly positive correlation with immune-related pathways, such as complement system, allograft rejection, and IL-2-STAT5 signaling (Figure 6K). In addition, PTGR1 had a positive correlation with the metabolic pathways and a negative correlation with the immune pathways (Figure 6M).

We also analyzed the correlation of these genes with immune cells and the expression of immune checkpoints. The analysis is illustrated in Figure 6N. It was worth noting that SH3KBP1 was positively correlated with immune checkpoints such as CD80, CD86, HAVCR2, PDCD1LG2, CTLA4, PDCD1 and CD274 (*r* = 0.55, 0.53, 0.52, 0.47, 0.47, 0.44, and 0.38, respectively). These results highlighted its role in the progression and prognosis of HNSC. Furthermore, there was a negative correlation between ABCC5 and PDCD1LG2 (*r* = -0.36), between AGTRAP and B and plasma cells (*r* = −0.28, −0.22), and between PACS2 and memory CD4 T cells and neutrophils (*r* = −0.23, 0.21). These genes could play a role in carcinogenesis and the tumor immune microenvironment.

## Discussion

HNSC is a diverse group of cancers occurring in the oral cavity, oropharynx, and larynx. The limitations of the anatomic region, TNM stage, and HPV status as predictors for prognosis and treatment outcomes have limited clinical applications. Genome-wide research revealed that HNSC patients can be categorized into separate AS subgroups according to their AS patterns, which exhibited uneven distribution of survival status, EGFR mutation/amplification, TP53 mutation, and immune characteristics (Li et al., 2019). These data indicated that AS events may help stratify high-risk patients and predict the treatment response. In this study, we used the LASSO-regularized Cox regression model to screen eight prognostic AS events and established a prognostic signature for HNSC. Patients can be easily classified as high- or low-risk, according to our signature. Our results reported that the high-risk group had a significantly worse survival outcome. The multivariate Cox regression analysis revealed that risk score was independent of clinical variables such as age, pathological stage, and HPV status. The ROC curve displayed the AUC of risk score above 0.75 in both the short- and long-term. These results highlighted the predictive capability of our signature.

Previously, a genome-wide AS profiling analysis revealed that clusters of AS with distinct patterns correlated with different immune statuses (Li et al., 2019). This was consistent with our finding that the AS risk score was connected to distinct immune cell populations and immune activation. The findings indicated that the predicted low-risk group had a higher level of immune cell infiltration (e.g., CD8^+^ T cells, Tregs, memory CD4 T cells, naïve B cells, and M1 macrophages) and immune activation signatures (e.g., cytolytic activity, CD8+/Treg ratio, and IFN-γ signaling). Conversely, most immune cells and activation signatures were lowly expressed in high-risk patients. However, we observed an increased infiltration of M2 and M0 macrophages in these patients. These results characterized low-risk patients as “Immune Class”, with high immune cell infiltration, enhanced cytolytic activity, and active IFN signaling (Mandal et al., 2016; Chen et al., 2019). In contrast, high-risk patients were labeled as “non-Immune Class”, with “Exhausted Subtype” characteristics such as increased M2 macrophage infiltration (Chen et al., 2019).

Anti-tumor immunity is dependent on several key aspects:1) recognition of tumor-specific antigens; 2) immune cell infiltration; 3) freedom from immunosuppressive effects of immunoregulatory pathways and cells (Kim and Chen, 2016). Non-synonymous mutation load, which can result in the expression of neoantigens, may contribute to immunogenicity and inflamed cancer phenotype (Goodman et al., 2017; Jardim et al., 2021). However, we did not find a difference in the mutation load between high- and low-risk groups. In addition to the neoantigens generated by mutations, viral antigens can compromise the immune system and are targeted by T-cells (Tashiro and Brenner, 2017). HPV + HNSC has a better prognosis than HPV-cancer, reflecting the high level of viral activity (Mandal et al., 2016; Gameiro et al., 2018; Johnson et al., 2020). Our research found that the proportion of HPV + HNSC was lower in the high-risk group, indicating that the difference in the HPV + cancer distribution may be part of the low immunity in the high-risk group. Antigens released by tumor cells are taken up by antigen-presenting cells (APC), such as dendritic cells (DCs), which become activated and migrate to the tumor-draining lymph node. The activation of DCs is known to initiate the cancer-immunity cycle (Steinman, 2012). In comparison with the low-risk group, DC signatures (e.g., activated and plasmacytoid DCs) were lowly expressed in the high-risk group, contributing to the low immune infiltration in these patients. For successful trafficking or infiltration, the immune cells need to overcome the pressure from the tumor itself and the surrounding stroma cell (Ariffin et al., 2014; Joyce and Fearon, 2015; Turley et al., 2015). Stromal cells can reduce lymphocyte adhesion by regulating the expression of adhesion molecules (e.g., ICAM-1 and VCAM-1) (Griffioen et al., 1996; Bouzin et al., 2007). Recent studies have shown that activated stromal cells combined with increased TGF-β and M2 infiltration can lead to an immunosuppressive phenotype. This correlated to poor prognosis and good response to immunotherapy (Chen et al., 2019). Nevertheless, no difference was found in stroma cell scores and extracellular matrix signatures between the two risk groups. This indicated that the exclusion exerted by stromal cells may not be the reason for the difference in immune infiltration between the two groups.

Anti-tumor immunity also relieves the immunosuppressive effects from immune pathways and regulatory cells. Our research demonstrated that in the low-risk group, most immune checkpoints were highly expressed, and some were negatively correlated with the risk score. These results are consistent with a previous observation that immune-high HNSC is often accompanied by a high immunoregulatory response (Mandal et al., 2016). Interestingly, we found a high expression of CD276 in the high-risk group. CD276, also known as B7 homolog three protein (B7-H3), is recently considered a T cell inhibitor that promotes tumor proliferation and invasion (Zhou and Jin, 2021). Compared to other immune checkpoints, CD276 affects immunity, as well as regulates the cancer cell aggressiveness *via* multiple non-immune pathways (Liu et al., 2011; Li et al., 2017; Shi et al., 2019). Overexpression of CD276 was found in various tumors, including HNSC, and conferred a poor prognosis (Liu et al., 2021b). Recent research reported a negative correlation between CD276 expression and CD8^+^ T cell infiltration in human HNSC (Mao et al., 2017). Inhibition of CD276 increased the infiltration of CD8^+^ T cells and T cell activation in mice models (Mao et al., 2017; Wang et al., 2021b). Given these results, the abnormal expression of CD276 might be responsible for excluding the immune cells in the high-risk group. Immune cells are an important source of immunosuppression. Our results found that M2 macrophage infiltration was increased in high-risk patients. M2 macrophages have been demonstrated to reduce tumor-infiltrating lymphocytes, especially CD8^+^ T cells, by reducing the expression of chemokines and promoting the production of extracellular matrix (Peranzoni et al., 2018; Pan et al., 2020). The depletion of M2 macrophages had been reported to restore CD8^+^ T cell migration and infiltration, and improve the efficacy of immunotherapies (Peranzoni et al., 2018). Therefore, high expression of M2 macrophages may led to low immune infiltration in the high-risk group.

Immune hot tumors are better recognized by the immune system and can trigger a better response to checkpoint therapies (Kim and Chen, 2016). Therefore, we hypothesized that checkpoint blockade may benefit low-risk HNSC patients, with high immune infiltration and immune checkpoint expression (e.g., PD-1 and CTLA-4). Tregs are also highly infiltrated in these patients, indicating that a combination of molecular antagonists (e.g., CTLA-4, CCR4, and STAT3 antagonist) can attain a better response rate (Selby et al., 2013; Sugiyama et al., 2013; Woods et al., 2018). In contrast, the immune landscape of the high-risk group is characterized by lower immune cell infiltration and higher expressions of CD276 and M2 macrophages. This kind of immunologically cold tumor can be responsive to the checkpoint blockade by a combination of therapies that promote tumor immune cell infiltration and convert tumors into an immunologically hot phenotype (Ribas et al., 2018). Therefore, we hypothesized that the strategy of combining the following therapies can be more effective in the high-risk group:

1) Cytokine and tumor vaccine therapies. A randomized phase III trial of IL-2 given *via* the perilymph after oral cavity surgery reported that cancer patients exhibited >25% improvement in OS (De Stefani et al., 2002). Furthermore, perilymphatic delivery of cytokines increased lymphocyte infiltration and improved prognosis in HNSC patients (Berinstein et al., 2012). Cancer vaccines can bypass the immune cold tumor microenvironment and deliver antigens directly to the APC (Tan et al., 2018; Shibata et al., 2021). This approach displayed significant potential to upregulate CD8^+^ T cells and sustain their function (Ott et al., 2017). HPV E6 and E7 are ideal vaccine targets for HNSC because of their highly immunogenic epitopes. Clinical trials assessing E6/E7 protein-based vaccines, DNA-based vaccines encoding E6/E7, or pathogen vector-based vaccines containing E6/E7-encoding DNA are currently in progress (Tan et al., 2018; Shibata et al., 2021).
2) Immunotherapy based on targeting CD276. CD276 expression is associated with resistance to anti-PD-1 immunotherapy in non-small cell lung and ovarian cancer (Yonesaka et al., 2018; Cai et al., 2020). In solid tumor mice models, blocking of CD276 with mAbs increased CD8^+^ T and NK cell infiltration, decreased tumor development, and prolonged survivability (Wang et al., 2021b). In addition to mAbs, chimeric antigen receptor (CAR) T cell technology is another option for targeted CD276 immunotherapy. Autologous T cells are designed with CARs to target tumor antigens and destroy cancer cells. Majzner et al. described a CAR-T cell system targeting CD276 which showed significant effectiveness against various xenograft cancer types, including osteosarcoma, medulloblastoma, and Ewing sarcoma (Majzner et al., 2019). They also demonstrated that the efficacy of CAR-T cells depended on the density of CD276 on the tumor surface. Recently, Tang et al. demonstrated that the local administration of CAR-T cells limited tumor development without off-target toxicity or major adverse effects in recurrent anaplastic meningioma (Tang et al., 2020). This evidence supports the application of CD276 CAR-T cell therapy in NHSC patients. Hence, future research should focus on this aspect.
3) Therapy to inhibit M2 macrophages. M2 macrophages, derived from peripheral blood monocytes, are recruited at the tumor site *via* the CCL2-CCR2 axis. Blockade of the CCL2-CCR2 axis could reduce the incidence of tumors by preventing M2 macrophage recruitment and enhancing the efficacy of CD8^+^ T cells in the tumor microenvironment (Yang et al., 2020). Targeting immunosuppressive molecules on M2 macrophages is also an effective method. CSF-1R, a tyrosine kinase transmembrane receptor on M2 macrophages, is currently the most studied tumor-associated macrophages (TAMs) (Peyraud et al., 2017). Several clinical trials have reported a decrease in M2 macrophages and an increase in CD8/CD4+T cell ratio in advanced solid tumors treated with single or combined anti-CSF-1R agents (Strachan et al., 2013; Ries et al., 2014; Peyraud et al., 2017). Other promising targets include MerTK, Axl, and Tyro3, which have yielded encouraging results in preclinical studies (Myers et al., 2019).

Finally, we performed expression and functional analysis on the parent genes in AS events. Our results highlighted the role of SH3KBP1 in tumor immune regulation in HNSC. SH3KBP1, also called Cbl-interacting 85 kD (CIN85), encodes an adaptor protein that is involved in many signaling pathways, connecting multiple cellular compartments and processes (Havrylov et al., 2010). We observed that the expression of SH3KBP1 was higher in tumors than in normal tissues, indicating a poor prognosis in HNSC. Importantly, our correlation analysis found a correlation between its expression and the immune checkpoint expression. Although SH3KBP1 mediates the inhibition of T cell activation (Kong et al., 2019), the regulatory mechanism remains unclear. Yakymovych et al. proved that SH3KBP1 promoted the expression of TGFβ receptor on the cell surface and positively regulated TGFβ signaling (Yakymovych et al., 2015). TGFβ signaling induced the expression of both PD-1 and PD-L1 in infiltrating T cells in multiple preclinical models (Baas et al., 2016; Strait and Wang, 2020). Furthermore, a recent study demonstrated that TGFβ induced PD-L1 *in vitro* on human non-small cell lung cancer cell lines by Smad2-mediated canonical TGFβ signaling (David et al., 2017). Therefore, TGFβ signaling may regulate the expression of immune checkpoints *via* SH3KBP1, which should be verified in future studies.

Several AS-based prognostic signatures have been identified in HNSC (Xing et al., 2019; Li et al., 2020; Zhao et al., 2020). However, these studies have not focused on the prognostic signatures and the tumor microenvironment. In this research, the AS events were analyzed in a large-scale HNSC cohort using the TCGA database. We established a predictive AS event signature for the prognosis and treatment of HNSC. This research had several limitations. Due to the lack of RNA-Seq data with survival information in the public database, we were unable to perform survival analysis in the independent cohort. Thus, these results need further validation by other available datasets and future research. Secondly, the two risk groups exhibited distinct immune landscapes, but their differences were insignificant. Hence, greater subgroup differentiation is required for precision therapy. Despite these limitations, our signature can successfully differentiate between high- and low-risk patients and suggest accurate treatment strategies.

## Conclusion

This research established a prognostic factor for HNSC patients according to the eight survival-associated AS events. The risk score was confirmed to be connected to HNSC prognosis and immune infiltration, suggesting that the prognostic signature could be an effective tool for the prognosis of HNSC patients and their clinical response to immunotherapy. However, this signature requires further research and validation in larger cohort studies.

## Data Availability

The original contributions presented in the study are included in the article/Supplementary Material, further inquiries can be directed to the corresponding author.

## Abbreviations

HNSC, head and neck squamous cell carcinoma; AS, alternative splicing; TCGA, the cancer genome atlas; SF, splicing factors; LASSO, least absolute shrinkage and selection operator; HPV, human papilloma virus; OS, overall survival; ROC, receiver operating characteristic; GSEA, gene set enrichment analysis; TMB, tumor mutational burden.
