# Supplementary material for: Construction of the prognostic signature of alternative splicing revealed the prognostic predictor and immune microenvironment in head and neck squamous cell carcinoma
Source: Front Genet. 2022 Oct 21;13:989081. doi: 10.3389/fgene.2022.989081 (PMC9633855; doi:10.3389/fgene.2022.989081)
Supplement: Supplementary file 1 [file Table1.DOCX]

**Table S1. The clinical characteristics of patients in the TCGA dataset**

| Characteristics | HNSC (N=486) |
| --- | --- |
| Age |  |
| Mean±SD | 61.01±11.94 |
| Follow up time |  |
| Median, days [min-max] | 564.00 [1, 6417] |
| Gender |  |
| Female | 131 (27.01%) |
| Male | 355 (72.99%) |
| Primary site |  |
| Alveolar ridge | 18 (3.71%) |
| Base of tongue | 23 (4.74%) |
| Buccal mucosa | 21 (4.33%) |
| Floor of mouth | 57 (11.75%) |
| Hard palate | 7 (1.44%) |
| Hypopharynx | 9 (1.86%) |
| Larynx | 108 (22.27%) |
| Lip | 3 (0.62%) |
| Oral cavity | 69 (14.23%) |
| Oral tongue | 123 (25.36%) |
| Oropharynx | 9 (1.65%) |
| Tonsil | 39 (8.04%) |
| Smoking history |  |
| Former and current smoker | 366 (75.26%) |
| Non-Smoker | 111 (22.89%) |
| unknow | 9 (1.86%) |
| Alcohol history |  |
| NO | 153 (31.55%) |
| YES | 322 (66.19%) |
| unknow | 11 (2.27%) |
| Pathologic T |  |
| T1 | 46 (9.28%) |
| T2 | 130 (26.80%) |
| T3 | 95 (19.59%) |
| T4 | 164 (33.81%) |
| TX | 33 (6.80%) |
| unknow | 18 (3.71%) |
| Pathologic N |  |
| N0 | 167 (34.23%) |
| N1 | 63 (12.99%) |
| N2 | 160 (32.99%) |
| N3 | 7 (1.44%) |
| NX | 69 (14.23%) |
| unknow | 20 (4.12%) |
| Pathologic stage |  |
| Stage I | 26 (5.15%) |
| Stage II | 69 (14.23%) |
| Stage III | 77 (15.88%) |
| Stage IV | 251 (51.75%) |
| unknow | 63 (12.99%) |
| HPV status |  |
| Negative | 271 (55.67%) |
| Positive | 60 (12.37%) |
| unknow | 155 (31.96%) |
| Progression |  |
| NO | 358 (73.61%) |
| YES | 83 (17.11%) |
| unknow | 45 (9.28%) |
| Dead |  |
| NO | 323 (66.39%) |
| YES | 163 (33.61%) |
