# Supplementary figures and images for: Construction of the prognostic signature of alternative splicing revealed the prognostic predictor and immune microenvironment in head and neck squamous cell carcinoma
Source: Front Genet. 2022 Oct 21;13:989081. doi: 10.3389/fgene.2022.989081 (PMC9633855; doi:10.3389/fgene.2022.989081)

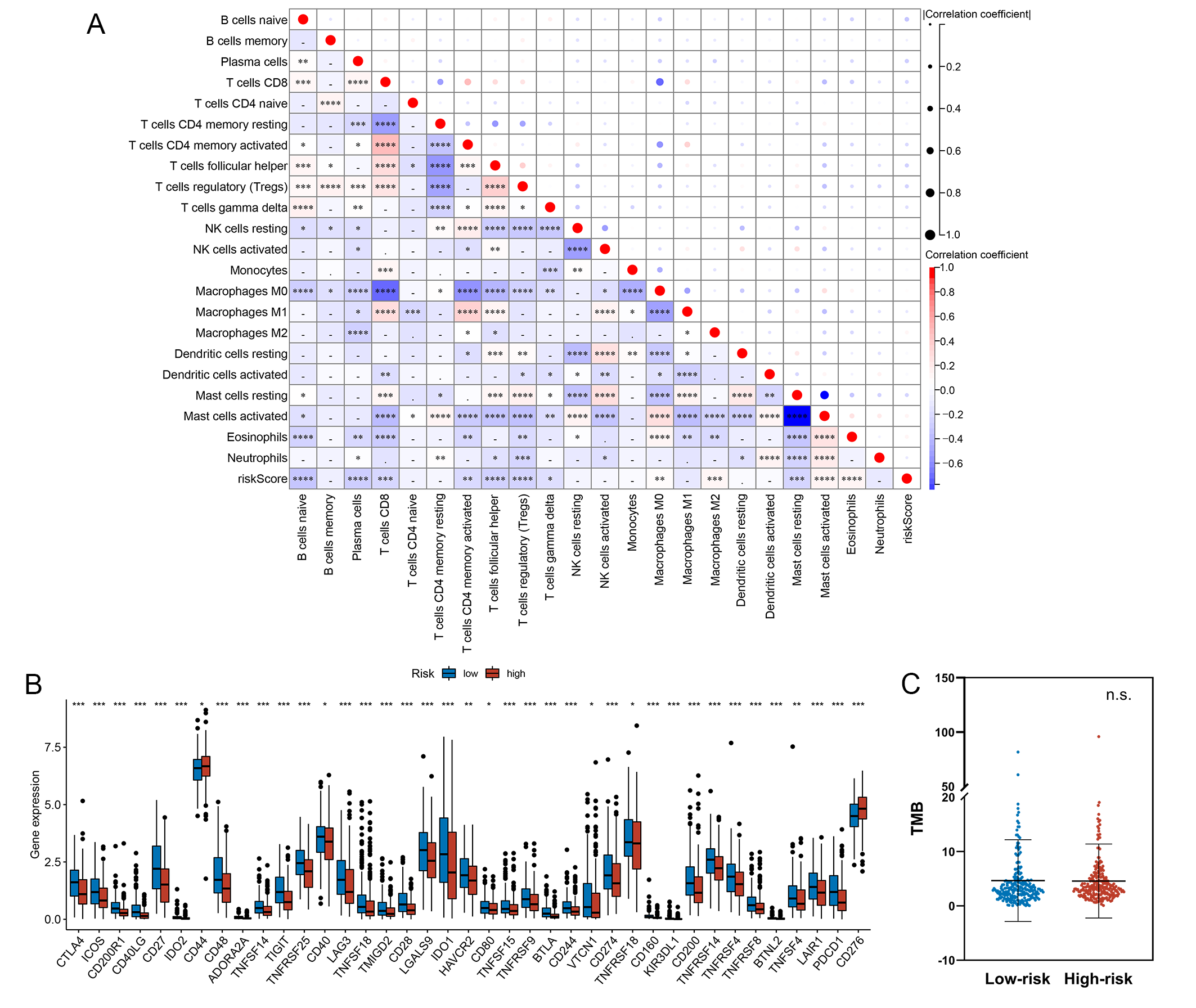

Supplement: Supplementary file 2 [file Image1.TIF]
