# Supplementary material for: Construction of the prognostic signature of alternative splicing revealed the prognostic predictor and immune microenvironment in head and neck squamous cell carcinoma
Source: Front Genet. 2022 Oct 21;13:989081. doi: 10.3389/fgene.2022.989081 (PMC9633855; doi:10.3389/fgene.2022.989081)
Supplement: Supplementary file 3 [file Table2.DOCX]

| **Alternative splicing event** | **Gene Description** | **Coefficient** | **HR** | **HR.95L** | **HR.95H** | ***P* value** |
| --- | --- | --- | --- | --- | --- | --- |
| AIG1\|77971\|AT | Androgen Induced 1 | -3.7660545 | 0.023143195 | 0.00117328 | 0.456502937 | 0.013309 |
| PACS2\|29633\|AP | Phosphofurin Acidic Cluster Sorting Protein 2 | -2.7058693 | 0.066812218 | 0.00447962 | 0.996485313 | 0.049702 |
| PTGR1\|87219\|AA | Prostaglandin Reductase 1 | -2.2007359 | 0.110721649 | 0.01315598 | 0.931840888 | 0.042876 |
| RHOT1\|40176\|ES | Ras Homolog Family Member T1 | -2.4611064 | 0.085340479 | 0.00995539 | 0.731563148 | 0.024762 |
| AGTRAP\|670\|AA | Angiotensin II Receptor Associated Protein | -1.9822401 | 0.137760289 | 0.03813979 | 0.49758784 | 0.002485 |
| ABCC5\|67820\|RI | ATP Binding Cassette Subfamily C Member 5 | -0.9756966 | 0.376929696 | 0.16069481 | 0.884135586 | 0.024893 |
| SH3KBP1\|88642\|AP | SH3 Domain Containing Kinase Binding Protein 1 | -1.4500882 | 0.23454961 | 0.09000325 | 0.611239266 | 0.003005 |
| RBMX\|90220\|RI | RNA Binding Motif Protein X-Linked | -1.0244199 | 0.359004678 | 0.15302111 | 0.842265224 | 0.018547 |

**Table S2. Information of survival‑related alternative splicing events involved in prognostic signature**

HR, hazard ratio; HR.95 L/H, 95% confidence interval of the hazard ratio.
